# Development of next-generation formulation against *Fusarium oxysporum* and unraveling bioactive antifungal metabolites of biocontrol agents

**DOI:** 10.1038/s41598-021-02284-1

**Published:** 2021-11-24

**Authors:** Monika Jangir, Shilpi Sharma, Satyawati Sharma

**Affiliations:** 1 Centre for Rural Development and Technology, Indian Institute of Technology Delhi, Hauz Khas, New Delhi, 110016 India; 2 Department of Biochemical Engineering and Biotechnology, Indian Institute of Technology Delhi, Hauz Khas, New Delhi, 110016 India

**Keywords:** Biochemistry, Biological techniques, Biotechnology, Microbiology

## Abstract

Biocontrol agents serve as a sustainable means of controlling wilt caused by the widespread plant pathogen, *Fusarium oxysporum* f. sp. *lycopersici*. The present study aimed to develop water dispersible granules (WDG) using response surface methodology (RSM) for *Bacillus subtilis* MTCC 2274 and *Trichoderma harzianum* MTCC 3928, and to compare their antifungal efficacy with other formulations. Further, characterization of the bioactive metabolites responsible for biocontrol was performed. A new microbial formulation, WDG, was developed in the present study with talcum powder (substrate), alginic acid (dispersing agent) and acacia gum (wetting agent) (suspensibility 82.23%; wetting time 2.5 min; dispersion time 10.08 min) that fulfilled the guidelines of Collaborative International Pesticides Analytical Council (CIPAC). *In planta* study demonstrated that WDG of *B. subtilis* showed maximum reduction in disease incidence (48%) followed by talc formulation of *B. subtilis* (44%) and WDG of *T. harzianum* (42%) with profound effect on plant growth promotion. *B. subtilis* and *T. harzianum* demonstrated protease (929 and 846 U ml^−1^ min^−1^), chitinase (33.69 and 154 U ml^−1^ min^−1^), and β-1,3-glucanase (12.69 and 21.47 U ml^−1^ min^−1^) activities. Culture filtrates of *B. subtilis* and *T. harzianum* exhibited significant inhibition against mycelial growth of pathogen. The compounds present in the culture filtrates were identified with GC–MS as fatty acids, alkanes, phenols, benzene, pyran derivatives etc. The major non-volatile compounds in bioactive antifungal fraction were identified as derivatives of morpholine and piperdine for *T. harzianum* and *B. subtilis*, respectively. The findings propose a multivariate biocontrol mechanism against phytopathogen by production of hydrolytic enzymes, volatile and non-volatile compounds, together with development of an efficient next-generation formulation.

## Introduction

Vascular wilt caused by *Fusarium oxysporum* f. sp. *lycopersici*, one of the most economically important disease in *Solanum lycopersicum*, is responsible for 10–90% loss in crop productivity^1^. It is evident that several *Fusarium* spp. have worldwide distribution, and economic significance as secretors of toxins including mycotoxins, fumonisins, secondary metabolites and other compounds causing infection in the host plant^2^. It is a soil-borne disease; pathogen starts infection with penetration from the roots and moves up to xylem of the plant^3^. The mycelia and spores of pathogen clog the vascular tissue of the plant. This causes stunting of the seedlings with yellowing and defoliation of older leaves, necrosis, loss of rigidity of non-woody parts of the plant, and browning of the xylem vessels that causes further infection in plants and ultimately leads to death of the plant^3,4^. Several methods viz., fumigation, solarization, crop rotation, mixed cropping, chemical fungicides etc. have been employed to manage this disease to minimize crop loss. The use of synthetic fungicides is effective but exerts a negative impact on the soil health and environment^5^. The prospects for the management of this soil borne pathogen using biocontrol agents has been extensively explored^6,7^.

A large number of biocontrol agents including bacteria and fungi such as *Bacillus, Trichoderma, Pseudomonas* have been thoroughly investigated. For the present study, *B. subtilis* and *T. harzianum* have been considered. *B. subtilis* is a gram-positive soil borne bacteria, which decreases or inhibits the harmful effects of the fungal pathogen through different mechanisms^7^. It serves the dual function of enhancing plant growth as well as exhibiting antagonistic activity against phytopathogens that could be beneficial in selecting possibly best biocontrol agent^8^. *B. subtilis* has some advantages over other biocontrol agents, such as tolerance to extreme environmental conditions which leads to the formation of resistant endospores^9^. Similarly, *T. harzianum* is also recognized as a potent fungal antagonistic against broad range of soil borne phytopathogens^7^. It produces hydrolytic enzymes, secondary metabolites, volatile compounds and antibiotics against plant pathogens^10,11^. Elucidating the nature of active compounds and their mechanism of action against pathogen could help in understanding the role of biocontrol agents in disease control. The current work focused on the isolation and identification of bioactive antifungal compounds produced by both the biocontrol agents that aid in mitigation of wilt disease.

To facilitate the application of a microbe-based product to the plant, the process of mass production of biocontrol agent, development of microbial formulation and its application should be taken into consideration. According to a previous study, development of efficient and practical delivery methods for the application of biocontrol agents to the soil ecosystem is a vital factor of biocontrol technology^12^. A formulation is considered as a stable and standardized mixture of inert and active components that results to obtain simple, safe and highly efficacious product to employ against target pathogen. The restoration of microorganisms using different carrier materials facilitates their easy-handling, longer shelf life, high efficiency and easy applicability in the fields^13^. Various types of formulation have been reported that include dusts, granules, pellets, wettable powders, capsules/beads, water dispersible granules or emulsifiable liquids. The powder formulation is not easy to weigh and apply in the field, and can cause health hazard to the applicator. The goal of present study was to develop a formulated product, which is user and environment-friendly. *B. subtilis* (MTCC 2274) and *T. harzianum* (MTCC 3928) were tested for their antagonistic efficacy against *F. oxysporum* in in vitro and in vivo studies conducted in previous study^14,15^. In the current study, next generation formulation of these strains was developed for easy application and storage. The antagonistic efficacy of the next-generation formulation developed in the study was evaluated in *in planta* assay under field conditions. To the best of our knowledge, present work is the first study on the development of microbial water dispersible granules, and characterization of the bioactive microbial metabolites against *F. oxysporum* f. sp. *lycopersici.* The present study demonstrated an approach to identify antifungal compounds of microorganisms responsible for biological control against phytopathogens.

## Results

### Development of WDG

**Figure 1:**
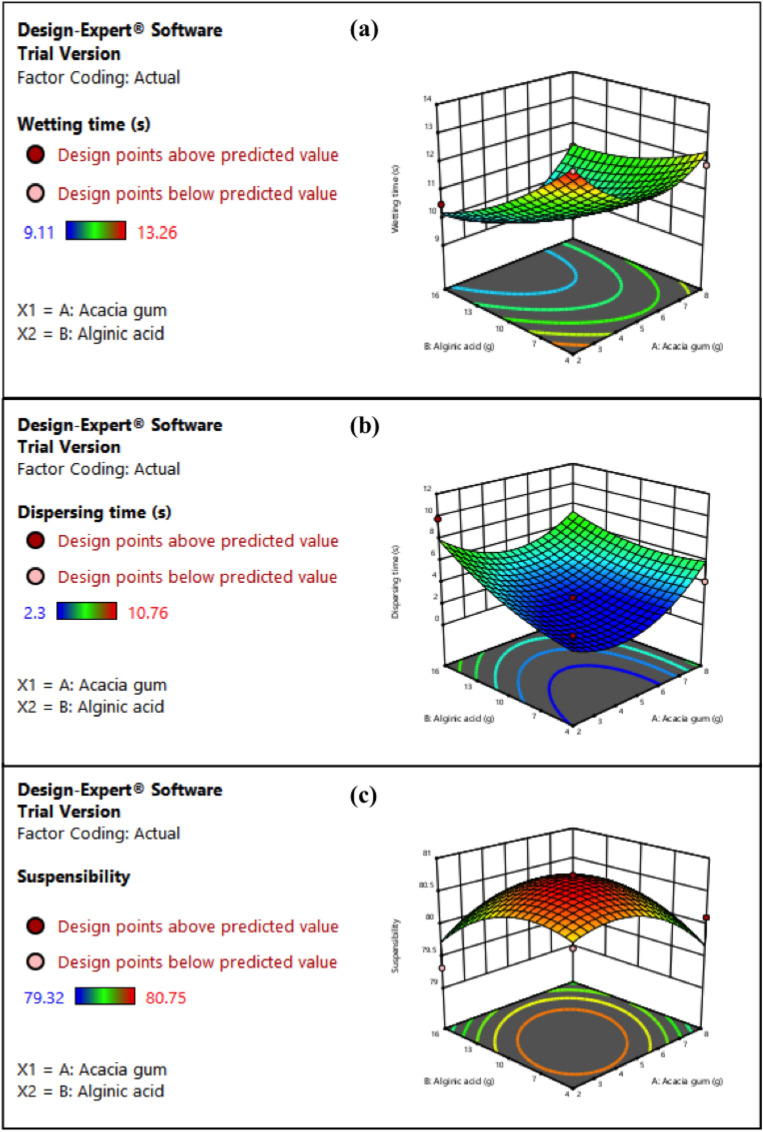
3D response curve showing interaction between concentrations of acacia gum-A and alginic acid-B on (**a**) wetting time, (**b**) dispersing time, and (**c**) suspensibility of water dispersible granules.

Two-step approach was followed for the development of efficient water dispersible granules. First step included selection of appropriate substrate, bulking agent, dispersing agent and wetting agent for developing WDG. The second step was optimization of the selected reagents by central composite design (CCD) using RSM. It was observed that combinations I, VI, and VII exhibited dispersion of granules in water (Table S1). In combination I, it was found that higher concentrations of napthalene sulphonate (> 1%) and sodium lauryl sulphate (> 5%) used as dispersing and wetting agents, respectively, inhibited the growth of both biocontrol agents in poisoned food technique (Table S2). Therefore, 1 and 5% of napthalene sulphonate and sodium lauryl sulphate was found optimum for development of WDG, respectively. Combination VI (acacia gum, talcum powder and alginic acid) showed highest dispersion rate among all the combinations (Table S1). Hence, it was further optimized in order to enhance the dispersing efficiency of obtained granules. For all combinations, no foaming was noticed. Further, experimental results for water dispersible granules using two-factor central composite design with five central points have been shown in Table 1. The efficiency of WDG was dependent on the concentration of factors viz., wetting agent (A) and dispersing agent (B). Statistical significance of the quadratic equation model evaluated by F-value and P-value for ANOVA of response 1 (wetting time), response 2 (dispersing time) and response 3 (suspensibility) have been presented in Table S3 (a-c, respectively). The model F-value for response 1 (wetting time), was 40.50, which depicted that the model was significant. There was only 0.01% chance that this large F-value could appear because of noise. It was noticed that the model terms were significant with P-values less than 0.05. Similarly, the model F-value (20.95 and 14.62) for response 2 (dispersing time) and response 3 (suspensibility), respectively, indicated that the both models were significant. Further, there were only 0.04 and 0.14% chances for response 2 and response 3, respectively, that this high F-value could appear due to noise. P-values for all responses were lower than 0.05 indicating that quadratic regression models were highly significant. The lack of fit for the model was insignificant as presented by F-values for all responses. The 3D response surface plots (Fig. 1) depicted the interaction between each independent variable viz., wetting and dispersing agents on wetting time, dispersing time and suspensibility of WDG. The slope of ridge for wetting time depicted that wetting time was directly proportional with concentration of acacia gum whereas increasing concentration of alginic acid reduced the wetting time (Fig. 1a) of WDG. However, increasing concentrations of factors viz., acacia gum and alginic acid reduced the suspensibility of granules (Fig. 1c) whereas dispersing time decreased with increasing concentration of alginic acid. As per RSM-CCD, run 9 showed the optimum conditions for the dispersal of WDG with 5 g of wetting agent (acacia gum) and 10 g of dispersing agent (alginic acid). The wetting time was recorded as 10.08 s without swirling and then the formulation instantly dissolved in 2.25 s without formation of any lump. The suspensibility revealed that 82.23% of granules was suspended in the water after 30 min. Hence, these optimum concentrations of acacia gum (5 g) and alginic acid (10 g) were selected to develop WDG and test in *in planta* assay. Figure S1 shows the dispersion of optimized microbial granules in water.

**Table 1:** Experimental design and results of central composite design for the development of efficient water dispersible granules.

Run	Variable A	Variable B	Response 1	Response 2	Response 3
	Wetting agent (g)	Dispersing agent (g)	Wetting time (s)	Dispersing time (s)	Suspensibility (%)
1	0.76	10	12.46	4.27	75.43
2	5	10	9.84	3.9	81.33
3	2	16	10.5	6.08	72.32
4	8	16	11.12	10.35	80.1
5	5	10	10.3	2.7	80.12
6	2	4	13.26	3.78	78.12
7	9.24	10	13.05	10.76	75.37
8	5	18.49	9.11	7.31	80.07
9	5	10	10.08	2.25	82.23
10	8	4	11.9	9.78	73.11
11	5	10	10.18	3.32	79.2
12	5	1.51	12.7	2.3	77.87
13	5	10	9.93	2.9	81.91

### In planta assay for efficacy evaluation of formulations in disease control

**Figure 2:**
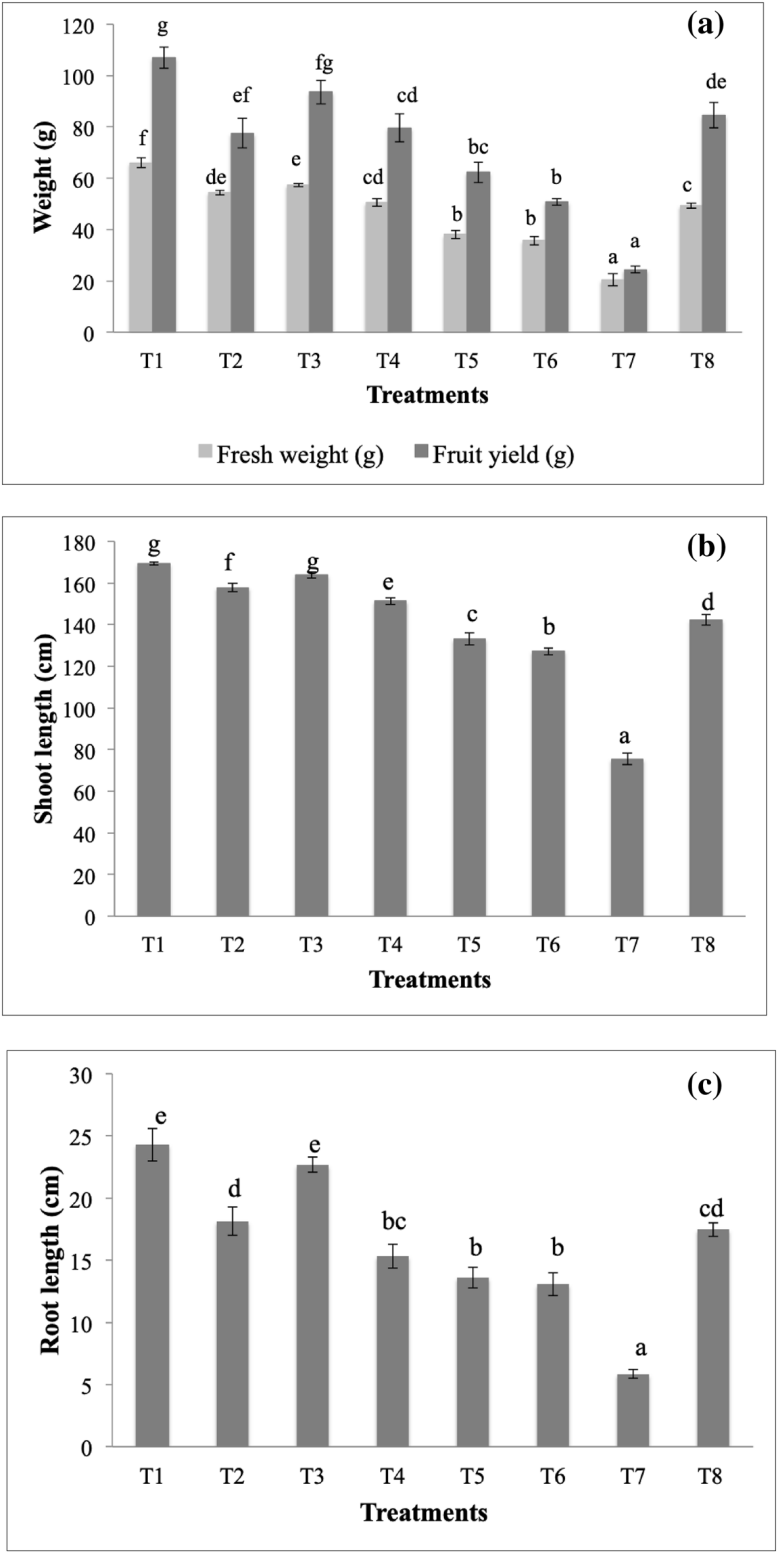
(**a**) Fresh weight and fruit yield, (**b**) shoot length and (**c**) root length of tomato plants treated with T1 = water dispersible granules (WDG) (*B. subtilis*) + *F. oxysporum*, T2 = WDG (*T. harzianum*) + *F. oxysporum*, T3 = Talc formulation (*B. subtilis*) + *F. oxysporum*, T4 = Talc formulation (*T. harzianum*) + *F. oxysporum*, T5 = alginate beads (*B. subtilis*) + *F. oxysporum*, T6 = alginate beads (*T. harzianum*) + *F. oxysporum*, T7 = only *F. oxysporum* and T8 = carbendazim (1000 ppm) + *F. oxysporum*. In each figure, bars with significantly different values by Tukey’s HSD test (*p* < 0.05) are marked with different letters. Error bars indicate standard deviation of triplicates.

Among all the treatments, best results were obtained with WDG of *B. subtilis* with maximum reduction in disease incidence (48%) followed by talc formulation of *B. subtilis* (44%) and WDG of *T. harzianum* (42%) (Table 2). Alginate beads of *T. harzianum* and *B. subtilis* were not capable of controlling disease efficiently and showed only 28% and 31% of disease reduction, respectively. With WDG of *B. subtilis*, increments of 3.16-fold, 1.25-fold, 2.24-fold and 3.38-fold in root length, shoot length, fresh weight and fruit yield, respectively, was observed (Fig. 2).

**Table 2:** Antifungal spectrum of microbial formulations in *in planta* assay under natural conditions to control *Fusarium* wilt in *Solanum lycopersicum.*

Treatment	Description	Disease reduction (%)	Disease severity index (%)
T1	WDG (*B. subtilis*) + *F. oxysporum*	48	43.3
T2	WDG (*T. harzianum*) + *F. oxysporum*	42	48.3
T3	Talc formulation (*B. subtilis*) + *F. oxysporum*	44	46.7
T4	Talc formulation (*T. harzianum*) + *F. oxysporum*	40	50
T5	Alginate beads (*B. subtilis*) + *F. oxysporum*	31	57.5
T6	Alginate beads (*T. harzianum*) + *F. oxysporum*	28	60
T7	Only *F. oxysporum*	-	83.3
T8	Carbendazim (1000 ppm) + *F. oxysporum*	51.9	40

*n* = 9; WDG = water dispersible granules.

### Hydrolytic enzyme activity

**Figure 3:**
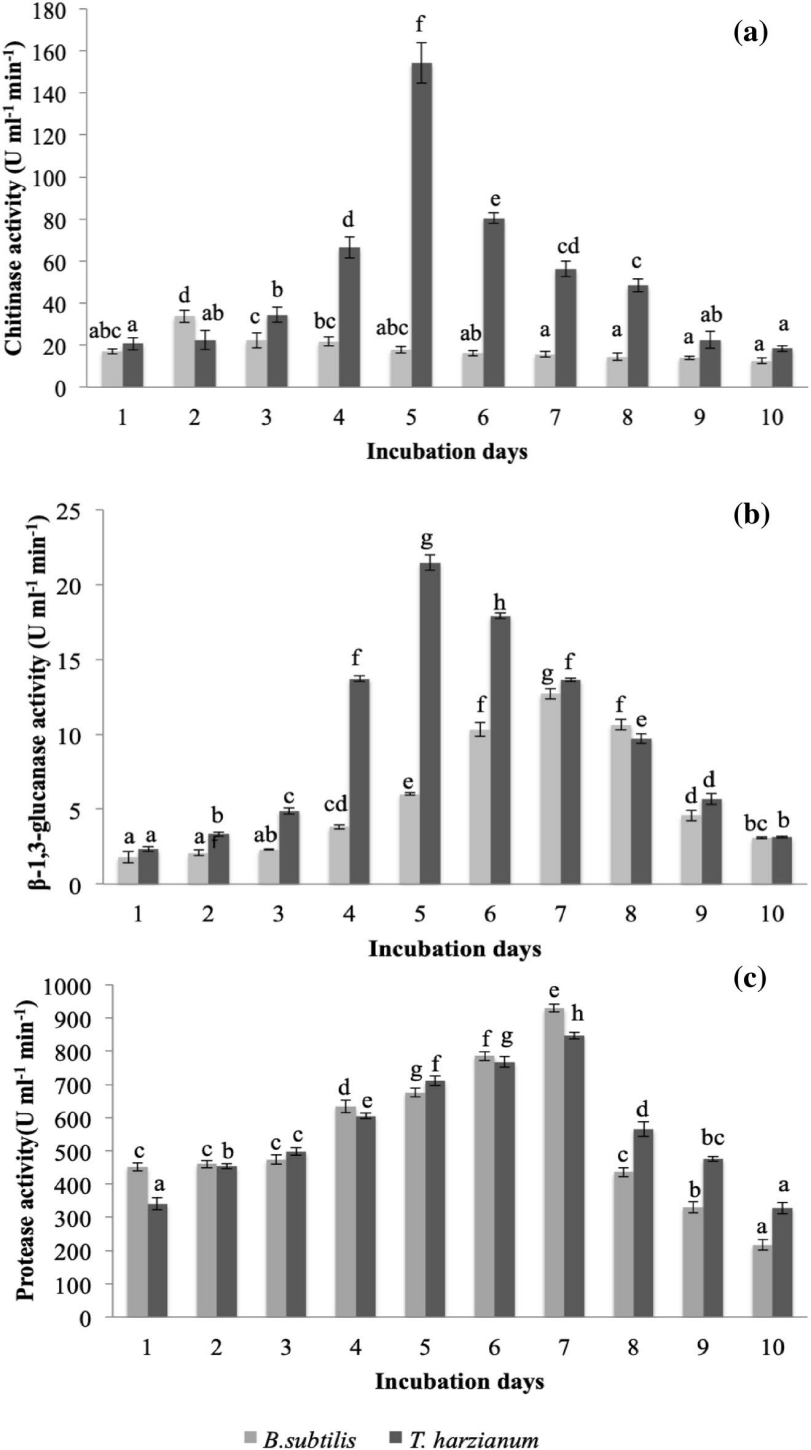
Production of (**a**) chitinase, (**b**) β-1, 3-glucanase and (**c**) protease by *Trichoderma harzianum* and *Bacillus subtilis*. In each figure, bars with significantly different values by Tukey’s HSD test (*p* < 0.05) for each biocontrol agent are marked with different lowercase letters. For each biocontrol agent, comparison has been done between enzymatic activities at every day. Error bars indicate standard deviation of triplicates.

Quantitative assays were carried out for estimating the potential of biocontrol agents in production of hydrolytic enzymes. It was noticed that incubation time affected the enzyme activity. It was apparent that among all the sampling days *T. harzianum* showed significantly highest chitinase activity (154.23 U ml^−1^ min^−1^) at 5 days after incubation (DAI) whereas *B. subtilis* exhibited highest value (33.69 U ml^−1^ min^−1^) at 2 DAI (Fig. 3). Thereafter, it declined gradually with time. Minimum activity of 12.47 and 18.43 U ml^−1^ min^−1^ was recorded at 10 DAI for *B. subtilis* and *T. harzianum*, respectively. Furthermore, *B. subtilis* and *T. harzianum* initially showed low activity of β-1,3-glucanase (2.3 and 1.8 U ml^−1^ min^−1^, respectively), which steadily increased to maximal value of 21.47 and 12.69 U ml^−1^ min^−1^, respectively. It was noted that enzymatic activity of β-1,3-glucanase was highest during the stationary phase of the growth, and similar trend was observed in the case of protease. For *T. harzianum* and *B. subtilis*, substantial increase was recorded in protease activity from 1 to 7 DAI exhibiting highest activity (846 and 929 U ml^−1^ min^−1^) after 7 DAI, respectively, among all the sampling days. Thereafter, a decline was observed to 327.66 and 216 U ml^−1^ min^−1^ , respectively, for *T. harzianum* and *B. subtilis* after 10 DAI. It was apparent that chitinase activity was maximum during exponential phase and subsequently decreased during the stationary phase.

### Activity of compounds produced by biocontrol agents in culture filtrate and identification by GC–MS

Culture filtrates of *T. harzianum* and *B. subtilis* at varying concentrations inhibited the mycelial growth of fungal pathogen. Percentage inhibition was more pronounced on increasing the concentration of microbial culture filtrate of biocontrol agents from 10 to 40% (Table 3). An inhibition of 44.66 and 58.51% was recorded with 40% culture filtrate of *T. harzianum* and *B. subtilis,* respectively (Table 3a, b). There was no significant difference in inhibition efficacy on further increasing the concentration of culture filtrate beyond 40%. GC–MS presented an extensive profile of 102 and 82 compounds for *B. subtilis,* and *T. harzianum,* respectively (Fig. S2). The five major compounds have been presented in Table 4, and their structures have been compiled in Fig. S3. Prominent compounds produced by *B. subtilis* and *T. harzianum* were 1-nonadecene (10.32%) and 6 pentyl-2H-pyran-2-one (12.3%), respectively.

**Table 3:** Percentage inhibition of culture filtrates (CF) of (a) *Trichoderma harzianum* and (b) *Bacillus subtilis* on radial growth of *Fusarium oxysporum*. In each column, significantly different values by Tukey’s HSD test (*p* < 0.05) are marked by different lowercase letters. Values are depicted with standard deviation for *n* = 3.

Incubation days	Percentage inhibition			
	10% CF	20% CF	30% CF	40% CF
**(a)**				
1	2.73 ± 0.62^a^	10.66 ± 0.78^ab^	11.23 ± 1.02^a^	18.64 ± 0.71^a^
2	4.83 ± 0.43^b^	10.68 ± 0.58^a^	15.27 ± 1.13^b^	22.37 ± 0.47^ab^
3	5.22 ± 0.59^b^	12.54 ± 0.78^b^	18.82 ± 0.65^c^	26.62 ± 0.63^bc^
4	5.64 ± 0.92^b^	14.6 ± 0.71^c^	20.99 ± 0.67^c^	30.62 ± 0.9^ cd^
5	6.29 ± 0.55^b^	16 ± 0.34^ cd^	25.55 ± 0.91^d^	35.57 ± 1.21^d^
6	8.92 ± 0.77^c^	17.21 ± 0.53^d^	28.57 ± 0.71^e^	38.35 ± 0.68^d^
7	10.69 ± 0.85^c^	19.41 ± 0.87^e^	37.42 ± 0.86^f^	44.66 ± 1.12^e^
**(b)**				
1	3.25 ± 0.62^a^	11.81 ± 0.62^a^	15.58 ± 0.92^a^	25.52 ± 0.57^a^
2	3.88 ± 0.25^ab^	14.22 ± 0.68^ab^	15.93 ± 0.57^b^	26.52 ± 1.27^a^
3	4.7 ± 0.43^bc^	16.66 ± 0.39^b^	19.6 ± 0.41^b^	32.87 ± 1.76^b^
4	5.48 ± 0.72^c^	17.95 ± 0.82^ cd^	21.25 ± 0.52^b^	37.69 ± 0.97^c^
5	7.67 ± 0.45^d^	19.2 ± 1.32^cde^	28.35 ± 1.88^c^	39.74 ± 1.07^c^
6	9.85 ± 0.42^e^	20.42 ± 1^de^	32.21 ± 1.41^d^	47.61 ± 1.3^d^
7	11.2 ± 0.36^f^	22.12 ± 1.91^e^	40.77 ± 1.23^e^	58.51 ± 1.26^e^

**Table 4:** Major compounds in culture filtrate of (a) *Bacillus subtilis* and (b) *Trichoderma harzianum* identified by GC–MS in ethyl acetate extract.

S. no	Compounds	Retention time (min.)	Area (%)	Chemical formula	Molecular weight (g/mol)
**(a)**					
1	Pyrrolo [1,2-a]pyrazine-1,4-dione, hexahydro-3-(2-methylpropyl)-, (3S-trans)	28.89, 29.39, 29.59 & 37.32	12.27	C_11_H_18_N_2_O_2_	210.28
2	1-heptacosanol	34.1, 37.48 & 40.81	7.9	C_27_H_56_O	396.73
3	1-hexadecene	21.88	6.22	C_16_H_32_	224.43
4	Octadec-1-ene	26.37	5.79	C_18_H_36_	252.49
5	1-tetradecanol	16.79	4.55	C_14_H_30_O	214.39
**(b)**					
1	6 pentyl-2H-pyran-2-one	16.58, 16.95 & 18.38	12.3	C_10_H_14_O_2_	166.22
2	Pyrrolo [1,2-a]pyrazine-1,4-dione, hexahydro-3-(2-methylpropyl)	26.56, 27.12, 28.55 & 29.22	11.23	C_11_H_18_N_2_O_2_	210.28
3	Benzeneethanol,4-hydroxy	17.59	9.66	C_8_H_10_O_2_	138.16
4	Pyrrolo [1,2-a]pyrazine-1,4-dione, hexahydro-3-(2-methylpropyl)-,(3S-trans)	28.94 & 29.16	9.36	C_11_H_18_N_2_O_2_	210.28
5	Phenol,2,4-bis(1,1-dimethylethyl)	19.66	7.86	C_14_H_22_O	206.32

### Isolation of bioactive compounds

Extraction of compounds in cell free culture filtrate of biocontrol agents was carried out with hexane, ethyl acetate and butanol depending on varying polarities. An effect of crude extract of *B. subtilis* (50 mg/ml)*,* extracted with ethyl acetate, on suppression of radial growth and strong inhibitory effect on aerial mycelia of pathogen was evident (Fig. S4), and similar observation was detected for *T. harzianum*. For both biocontrol agents, six solvents with different polarities viz., ethyl acetate, petroleum ether, methanol, acetone, hexane and chloroform, were used as mobile phase for developing thin layer chromatography (TLC). Resolution of bands on TLC sheet developed with all solvents depicted the presence of compounds of varying polarities for *B. subtilis* and *T. harzianum* (Figs. S5 and S6, respectively). Among all solvents, ethyl acetate efficiently resolved the bands of *B. subtilis* crude extract on TLC sheet. For *T. harzianum*, several bands on TLC sheet developed with chloroform (CHCl_3_), which illustrated the presence of medium to low polar compounds (Fig. S6). However, petroleum ether (PE) also showed 2 bands that represented the presence of high polar compounds in the crude extract of *T. harzianum.* Therefore, combinations of these two solvents (CHCl_3_: PE) were further analyzed in different ratios from 1:9 to 9:1 to observe the pattern of band separation. Among all the combinations, mixture of CHCl_3_ and PE in the ratio of 8:2 was apparently suitable for developing TLC sheet. Then, in direct bioautography of *B. subtilis,* the white inhibition zone was observed at two spots ('a' and 'b') with Rf value of 0.625 and 0.475, respectively (Fig. S7). In a similar manner, two white spots (‘a’ and ‘b’) were observed for *T. harzianum* that represented the inhibition of *F. oxysporum* caused by bioactive compounds (Fig. S8). Retention factors (Rf) for bands ‘a’ and ‘b’ were calculated as 0.4125 and 0.175, respectively. All four bioactive bands were scrapped along with silica for in vitro agar well-diffusion assay as described above to evaluate their efficacy against pathogen. For *B. subtilis,* the inhibition zone obtained with band ‘b’ was higher than band ‘a’ and vice versa for *T. harzianum.* Hence, filtered fraction of band ‘b’ (Rf = 0.475) and band ‘a’ (Rf = 0.4125) was subjected to UPLC-MS for identification of antifungal metabolites.

**Figure 4:**
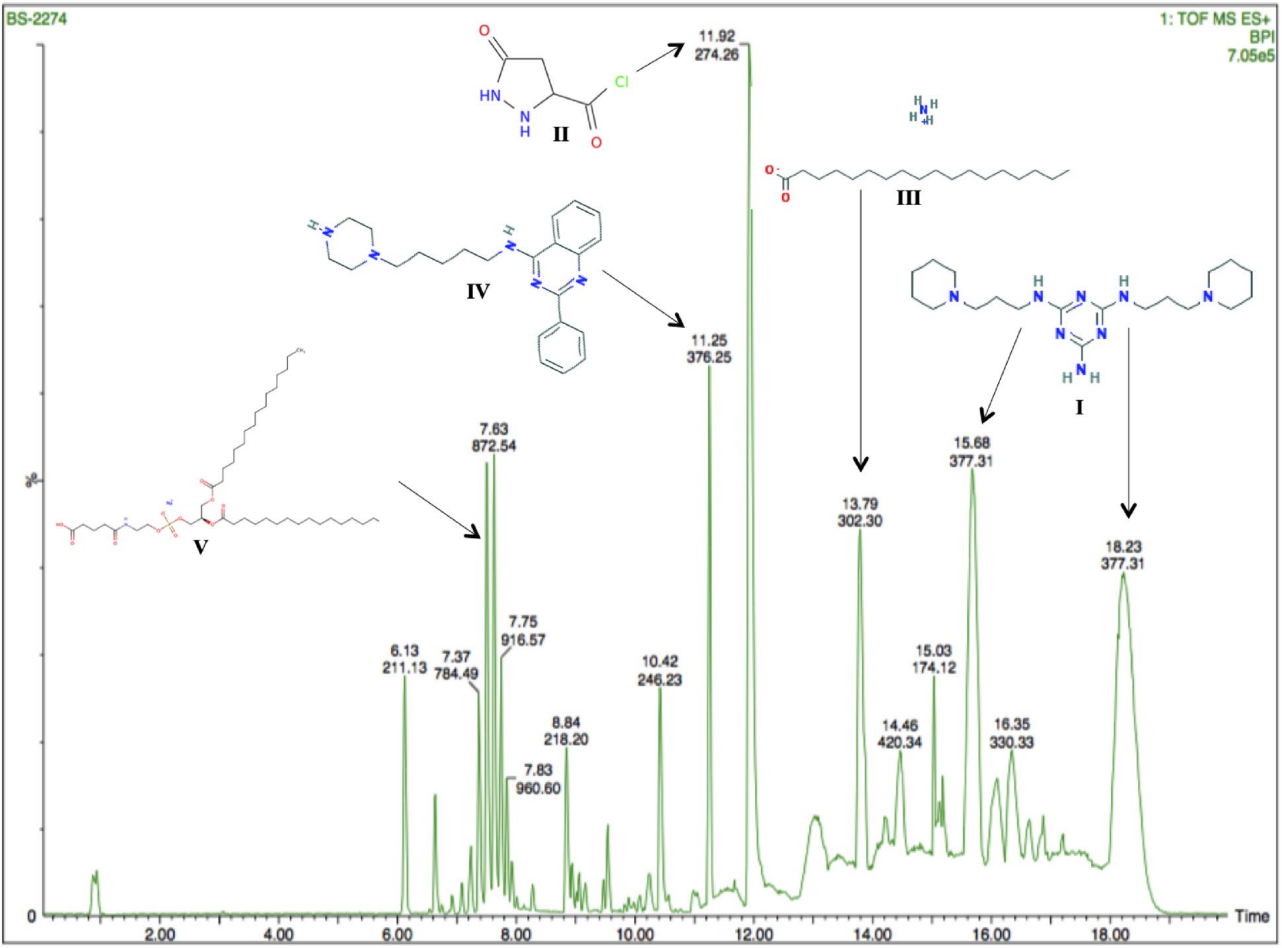
UPLC-MS chromatogram of bioactive fraction of ethyl acetate extract of *Bacillus subtilis* 2274 with retention time (min) on x-axis and area (%) on y-axis with chemical structure of major five compounds. For name of the compounds refer Table 5a.

**Figure 5:**
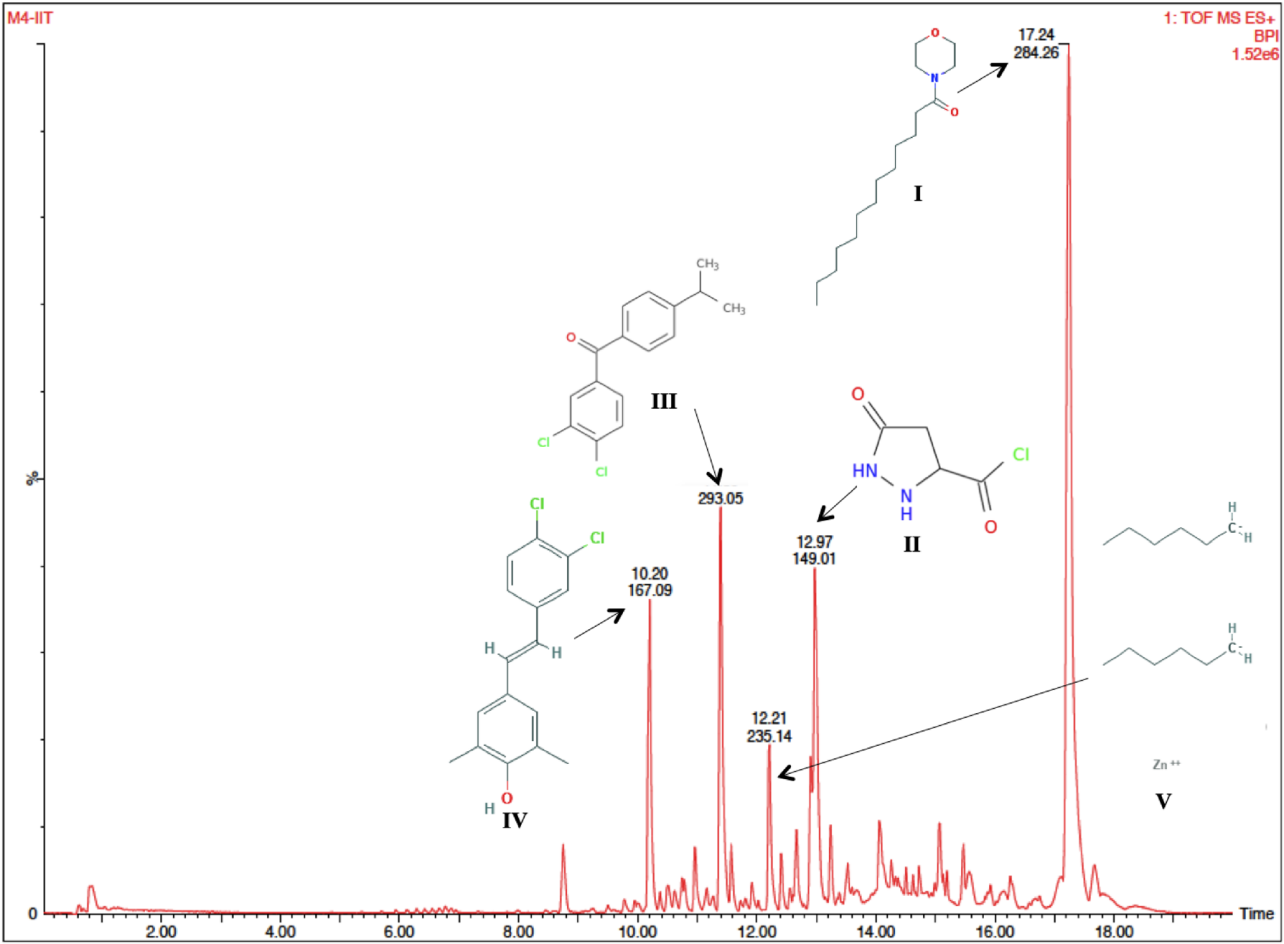
UPLC-MS chromatogram of bioactive fraction of ethyl acetate extract of *Trichoderma harzianum* 3928 with retention time (min) on x-axis and area (%) on y-axis with chemical structure of major five compounds. For name of the compound refer Table 5b.

For *B. subtilis,* a number of peaks were observed in UPLC-MS analysis of band ‘b’ (Fig. 4) and five major compounds were identified as compound I: N ~ 2 ~ ,N ~ 4 ~ -Bis[3-(piperidin-1-yl)propyl]-1,3,5-triazine-2,4,6-triamine, compound II: 1-[(RS)-3-(4-tert-butylphenyl)-2-methylpropyl]piperidine, compound III: octadecanoic acid, ammonium salt, compound IV: 2-phenyl-*N*-[5-(piperazin-1-yl)pentyl]quinazolin-4-amine and compound V: sodium (2R)-2,3-bis(hexadecanoyloxy)propyl 2-(4-carboxybutanamido)ethyl phosphate (Table 5a) and their structures are shown in Fig. 4. The descriptive UPLC-MS chromatogram for the fraction of band ‘a’ of *T. harzianum* is shown in Fig. 5. Five prominent peaks were evident in chromatogram suggesting major bioactive compounds, which might be responsible for its antagonistic activity against test pathogen (*F. oxysporum*). METLIN database identified these compounds as, compound I = morpholine, 4-tridecanoyl, compound II = 5-oxopyrazolidine-3-carbonyl chloride, compound III = 3,4-dichloro-4′-*n*-propylbenzophenone, compound IV = 4-[2-(3,4-dichlorophenyl)ethenyl]-2,6-dimethylphenol and compound V = Zinc, dihexyl (Table 5b) and their structures have been presented in Fig. 5.

**Table 5:** Major compounds identified by UPLC-MS in bioactive fraction of ethyl acetate extract of (a) *Bacillus subtilis* and (b) *Trichoderma harzianum.*

Compound	Name	Retention time (min)	m/z	Area (%)	Chemical formula
**(a)**					
**I**	N ~ 2 ~ ,N ~ 4 ~ -Bis[3-(piperidin-1-yl)propyl]-1,3,5-triazine-2,4,6-triamine	15.68 & 18.23	377.31	18.07	C_19_H_36_N_8_
**II**	1-[(RS)-3-(4-tert-butylphenyl)-2-methylpropyl]piperidine	11.92	274.26	15.24	C_19_H_31_N
**III**	Octadecanoic acid, ammonium salt	13.79	302.3	6.2	C_18_H_39_NO_2_
**IV**	2-phenyl-*N*-[5-(piperazin-1-yl)pentyl]quinazolin-4-amine	11.25	376.25	4.02	C_23_H_29_N_5_
**V**	Sodium (2R)-2,3-bis(hexadecanoyloxy)propyl 2-(4-carboxybutanamido)ethyl phosphate	7.5	828.52	3.66	C_42_H_79_NNaO_11_P
**(b)**					
I	Morpholine, 4-tridecanoyl	17.24	284.26	13.5	C_17_H_33_NO_2_
II	5-oxopyrazolidine-3-carbonyl chloride	12.97	149.01	10.17	C_4_H_5_N_2_O_2_Cl
III	3,4-dichloro-4′-*n*-propylbenzophenone	11.39	293.05	9.56	C_16_H_14_Cl_2_O
IV	4-[2-(3,4-dichlorophenyl)ethenyl]-2,6-dimethylphenol	10.2	167.09	7.45	C_16_H_14_Cl_2_O
V	Zinc, dihexyl	12.21	235.14	3.89	C_11_H_20_N_2_O_2_

## Discussion

The identification of bioactive metabolites, and understanding their biocontrol mechanisms is critical for the development of a successful biocontrol agent. In addition, commercial success of the biocontrol agent depends on the formulation of a stable and efficient product. The present study was conducted to develop new-generation formulation and to identify the bioactive compounds contributing to antagonistic activity of *T. harzianum* and *B. subtilis*.

Using RSM-CCD, the optimum concentrations of acacia gum (wetting agent) and alginic acid (dispersing agent) were determined, which resulted in the development of an efficient WDG. For an efficient WDG, the factors taken into consideration were wetting time, dispersing time and suspensibility. The optimized granules attained the wettability and start dispersing in minimum time. The suspensibility was maximum which implies that the components were not settling down at the bottom. Further, there was no foaming or frothing.* In* *planta* assay with optimized WDG revealed that efficacy of WDG is highest followed by talc formulation and alginate beads. A study was conducted with talc formulation, alginate beads and fresh culture of *T. harzianum* and *Pseudomonas fluorescens* to control *Fusarium* wilt in *Cajanus cajan*^16^*.* They reported wilt disease incidence of 31 and 20% with talc formulation as compared to 30 and 38% with alginate beads of *T. harzianum* and *P. fluorescens*, respectively, which concurs with the finding of the present study. It was found that WDG had a profound effect on plant growth promotion. This could be because of alginic acid, which might serve as a soil conditioner and fertilizer. Further, it has been reported that salts of alginic acid combine with metallic ions present in soil, and form complexes of high molecular weight that could improve crumb structure of soil by retaining moisture^17^. This enhances the capillary activity and soil aeration that further aids in stimulation of soil microbiome and boost plant growth.

**Figure 6:**
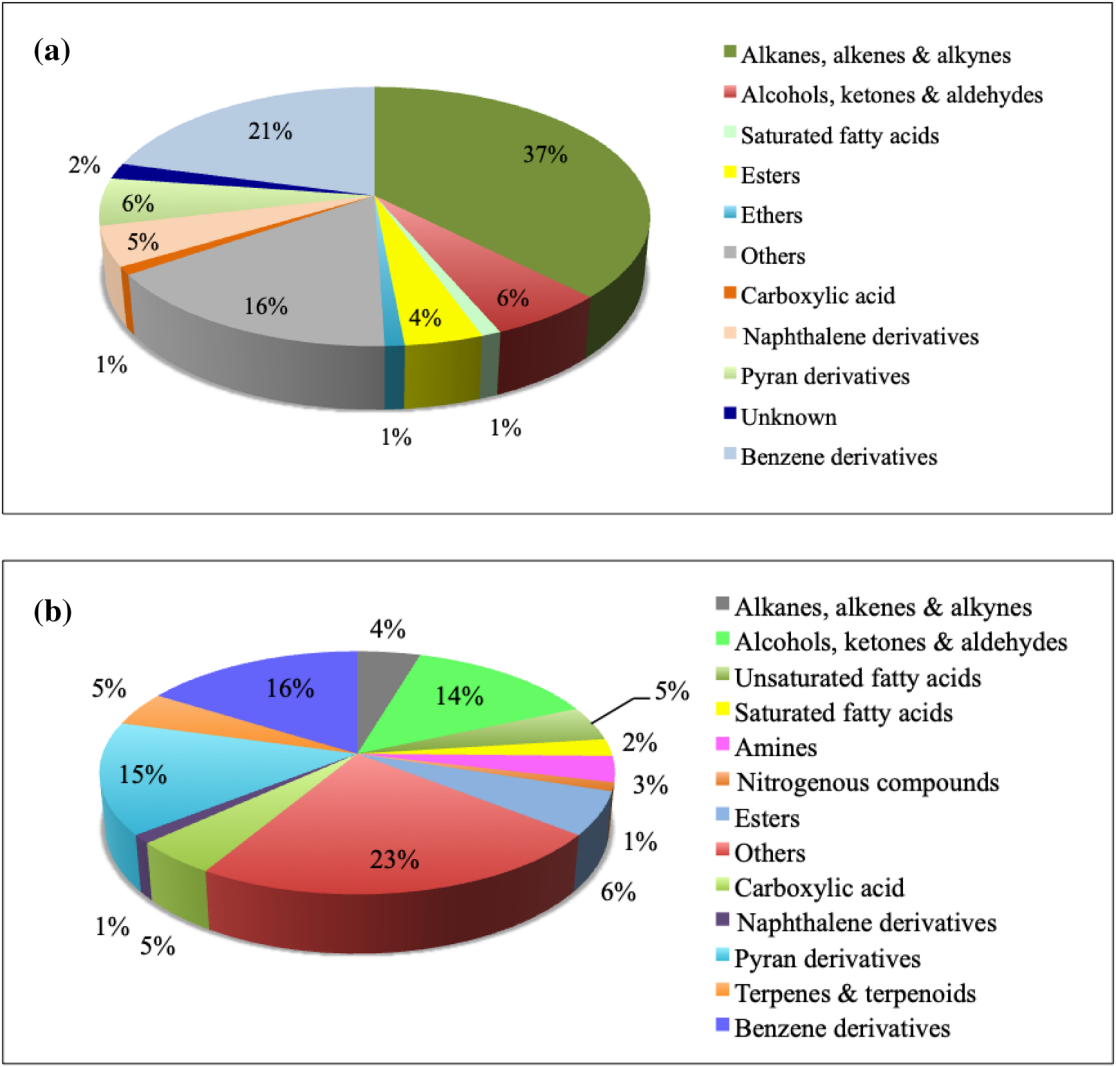
Percentage distribution of organic compounds in culture filtrates of (**a**) *Bacillus subtilis*, and (**b**) *Trichoderma harzianum*, identified by GC–MS in ethyl acetate extract.

The inhibition of fungal pathogen by microbial culture filtrate in the present study indicated that the mode of action of both biocontrol agents involved the secretion of antifungal secondary metabolites. It is important to mention that antagonistic efficacy of *B. subtilis* was more pronounced, which could be because of the production of a large number of aliphatic hydrocarbons (alkanes, alkenes and alkynes and their analogs) with 37% of the total compounds (Fig. 6). Aliphatic hydrocarbons could have lethal effect on pathogens; toxicity of the compound is directly correlated to its chain length^18^. Moreover, it was noticeable that benzene derivatives occupied a significant percentage (21%) of the total compounds produced by *B. subtilis*. Tang et al. reported the antagonistic activity of benzene derivatives against fungal phytopathogens viz., *F. oxysporum*, *Gibberella zeae*, *Phytophthora infestans* etc.^19^. It was clear that *B. subtilis* exhibited alcohol production (6%), which could increase the antagonistic efficacy against pathogen. The present findings are supported by earlier studies that include unraveling antimicrobial mechanisms of some organic solvents that get accumulated in cell membrane leading to the disorganization of structure and loss of membrane integrity^20^. Further, it results in loss of intracellular metabolites and ions that ultimately leads to pathogen cell death.

In the present study, a predominant compound in GC–MS analysis of *T. harzianum* was 6-pentyl-2H-pyran-2-one (6-PP) (12.3%) that is a chief antifungal compound synthesized by *Trichoderma* sp.^21^. Garnica-Vergara et al. revealed the role of 6-PP explaining the novel mechanism of enhancing root branching and plant growth^21^. The compound’s antagonistic role against *Botrytis cinerea*, *F. oxysporum* and *Rhizoctonia solani* has been established^22–24^*.* Previously researchers have reported compounds of similar category in their study of *Trichoderma* sp. namely, analogs of benzene ethanol, pyrone, pyrazine and phenol,2,4-bis(1,1-dimethylethyl) which is in accordance with the present study^25,26^.

Subsequently, an extensive experiment was carried out to isolate bioactive non-volatile compounds using direct bioautography and identified with UPLC-MS. For *B. subtilis,* compound I was identified as N ~ 2 ~ ,N ~ 4 ~ -bis[3-(piperidin-1-yl)propyl]-1,3,5-triazine-2,4,6-triamine, an analog of piperidine. Li et al. demonstrated the efficacy of piperidine against *Pythium ultimum* for controlling damping off of cucumber^27^. In addition, triazine moiety was also found in compound I associated with piperidine. A novel compound of triazine-based piperidine was synthesized and reported for its antagonistic activity against fungal pathogens viz., *Candida albicans* and *Aspergillus niger*^28^*.* Compound II was found to be 1-[(RS)-3-(4-tert-butylphenyl)-2-methylpropyl] piperidine, IUPAC name fenpropidin, which is a commercial fungicide. Its mode of action involves inhibition of sterol biosynthesis in membranes^29^. Compound III was identified as octadecanoic acid, ammonium salt, which is a saturated fatty acid derivative. Fatty acids have been reported for their antifungal activity against several phytopathogens^30,31^. Shelat and Vashi synthesized and tested the antifungal activity of a quinazolinone analog namely 2-(4-phenylpiperazinyl)methyl-3-(8-quinolinol-5-yl)-4(3h)-quinazolinone against several plant pathogens such as *Botrydepladia thiobromine*, *Penicillium expansum* and *Nigrospora* sp^32^. The compound synthesized by them showed similarity to compound IV namely, 2-phenyl-*N*-[5-(piperazin-1-yl)pentyl]quinazolin-4-amine identified in the current study. The antifungal activity of compound IV against fungal pathogen was supported by earlier studies reporting the antifungal activity of quinazolines analogues against *Phytophthora capsici*, *Colletotrichum gloeosporioides*, *Valsa mali*, and *Alternaria alternata*^33,34^. Compound V was identified as an aliphatic compound, sodium (2R)-2,3-bis(hexadecanoyloxy)propyl 2-(4-carboxybutanamido)ethyl phosphate, with long chain of 42 carbons; the toxicity of aliphatic compounds has been discussed earlier.

In the case of *T. harzianum*, compound I was identified as a morpholine analog i.e., morpholine, 4-tridecanoyl. Morpholine is a known commercial fungicide. Several researchers have evaluated morpholine analogs for antagonistic activity against phytopathogens such as *Aspergillus niger*, *A. clavatus*, *Botrytis cinerea, Candida krusei*^33,34^. Morpholine fungicides restrict the growth of pathogens by inhibiting sterol biosynthesis. Compound II was found to be a pyrazole analog, 5-oxopyrazolidine-3-carbonyl chloride. Pyrazole moiety is considered as an important class of heterocyclic compounds owing to its wide spectrum of biological activities including antifungal activity^35^. Previous studies assessed the antifungal efficacy of pyrazole analogues against *F. oxysporum* f. sp. *albedinis*^36,37^, which supported the antifungal activity of 5-oxopyrazolidine-3-carbonyl chloride identified in the present study against *F. oxysporum*. The third potent compound was identified as 3,4-dichloro-4′-*n*-propylbenzophenone. In a previous study, Al-Ghorbani et al. conducted in vitro bioassay for evaluating the efficacy of benzophenone analogues against *C. albicans, A. niger, F. solani, B. cinerea* and *C. krusei*, which supported the findings of the present study^38^. Compound IV was 34-[2-(3,4-dichlorophenyl)ethenyl]-2,6-dimethylphenol. Phenols play pivotal role in antifungal activity of the biocontrol agent. Compound V produced by *T. harzianum* was found as Zinc, dihexyl. It is an aliphatic hydrocarbon with possible lethal effect on pathogen as discussed previously.

Based on the findings of the current study, antifungal activity of biocontrol agents could be ascribed to production of bioactive secondary metabolites. The hydrolytic enzymes are required for the entry of antifungal compounds or antibiotics into the phytopathogen^39^. Biocontrol agents used in the current study are capable in production of hydrolytic enzymes (viz., chitinase, protease and β-1,3-glucanase). Recently, numerous species of *Bacillus* and *Trichoderma* have been reported for secretion of chitinase against mycotoxin producing fungi and phytopathogens by degrading the principal constituent of fungal cell wall^40,41^. Chitinases are also involved in inducing defense mechanism of the plants^41^. Other researchers have also investigated *B. subtilis* and *T. harzianum* for protease and β-1,3-glucanase activity against *F. oxysporum* which supports the findings of the present study^6,42–44^. Protease aids in degradation of proteinaceous content of the fungal cell wall and cytoplasmic proteins, whereas β-1,3-glucanase is involved in destroying pathogen cell wall by digesting glucans.

In the recent years the augmented burden on farmers for increased yield has resulted in excess usage of fertilizers and chemical pesticides. This has made it imperative to switch to environment friendly techniques. The present study suggested the application of biocontrol agents to control wilt disease in *S. lycopersicum.* A new efficient product i.e., water dispersible granules, was formulated for biocontrol agents exhibiting more disease reduction and enhanced plant growth owing to alginic acid present in the formulation. Recent investigations postulated that the production of protease, β-1,3-glucanase and chitinase might play a significant role in antagonistic activity of both biocontrol agents by degrading pathogenic cell wall.

## Methods

### Microorganisms

The cultures of *B. subtilis* (MTCC 2274) and *T. harzianum* (MTCC 3928) were procured from Microbial Type Culture Collection and Gene Bank, Institute of Microbial Technology (MTCC, IMTECH), Chandigarh. The fungal pathogen, *Fusarium oxysporum* f. sp. *lycopersici* (ITCC 1322), was procured from Indian Type Culture Collection, Indian Agricultural Research Institute (ITCC, IARI), Pusa, New Delhi. Bacterial strain was grown in Luria–Bertani broth (LB) at 30 °C for 2 days, and fungal strains were revived in potato dextrose broth (PDB) at 28 °C after incubation of 7 days. All the methods were carried out in accordance with relevant guidelines and regulations.

### Formulation development

The bacterial and fungal strains were cultured in LB and PDB under shaking condition of 120 rpm in orbital shaker (Orbitek, Scigenics Biotech, India) for 5 and 10 days at 30 °C, respectively^45^. The microbial biomass was removed from the broth by filtering through muslin cloth. Then, the filtered broth was used as an active ingredient to develop three types of microbial formulation. The methodology described by Jeyarajan et al. was followed to prepare talc formulation of fungal and bacterial biocontrol agents which has been explained in previous study^15,46^. Alginate beads with encapsulated microbial culture were prepared using the emulsification method under aseptic conditions^47^. Hundred ml of filtered fungal (10^8^ to 10^9^ spore ml^−1^) and bacterial broth (10^10^ to 10^11^ CFU ml^−1^) was mixed with 2 g of sodium alginate and kept for mixing on magnetic stirrer (Remi, India) at room temperature till homogeneous mixture was obtained (Fig. S9). The homogeneous mixture produced was added to CaCl_2_ solution (0.1 M) using sterile syringe (10 ml). As drop of the emulsion reacted with CaCl_2_, soluble sodium alginate was converted into water insoluble calcium alginate beads and NaCl was produced as by-product. The obtained microbeads were left overnight for air-drying in laminar airflow and then stored in sterile closed jars at 4 °C. For water dispersible granules, the selection of suitable chemical reagents was carried out as per the guidelines of Collaborative International Pesticides Analytical Council (CIPAC). All reagents (dispersing agent, wetting agent, bulking agent and substrate) were autoclaved and tested in vitro for any inhibitory effect towards the biocontrol agents using the poisoned food technique^48^.Then, 40 ml of culture (10^10^ cell ml^−1^) was adsorbed on 80 g substrate. For 100 g of formulation, adsorbed substrate (50 g) was mixed with dispersing agent (1 g), wetting agent (5 g) and bulking agent (44 g). It was mixed well by adding autoclaved water (15–20%) and then the paste was manually passed through an extruder machine of 0.3 mm mesh screen. After overnight air-drying the product was broken in granules and tested for its dispersing efficiency by adding in water. A number of combinations were tested for the development of WDG (Table S1) and the efficient one was further optimized to enhance the dispersion. The optimization analysis was undertaken to deduce the effect of individual components on the dispersing efficacy of WDG using the software Design Expert 11.1.0 (Stat-Ease, Inc., Minneapolis, USA). A central composite design was employed to determine the effects of two independent variables viz., alginic acid (dispersing agent, range: 4–16%) and acacia gum (wetting agent, range: 2–4%), and their interaction on the dispersing, wetting, foaming and suspensibility of WDG. The effect of each variable was considered at five different levels and was designated as − α,  − 1, 0, + α, + 1. According to this design with five central points, a total of 13 experiments were conducted with variable A as factor “wetting agent” and variable B as factor “dispersing agent”. The full experimental plan along with coded value of variables has been mentioned in Table S4.

### In planta assay to evaluate efficacy of formulations in wilt control under natural conditions

The three formulations (talc-based formulation, alginate beads and WDG) were evaluated for their efficiency in wilt control in *in planta* assay with a susceptible variety of *S. lycopersicum* (var. Pusa Ruby) under natural conditions. Seeds were collected from IARI, Pusa, New Delhi. Pusa Ruby is a commercial variety released by IARI and is susceptible to *F. oxysporum*. The assay was carried out in earthen pots with capacity of 4 kg soil. The experimentation was done in net house without light and temperature control, with the following treatments: T1 = WDG of *B. subtilis* + *F. oxysporum*, T2 = WDG of *T. harzianum* + *F. oxysporum*, T3 = talc formulation of *B. subtilis* + *F. oxysporum*, T4 = talc formulation of *T. harzianum* + *F. oxysporum*, T5 = alginate beads of *B. subtilis* + *F. oxysporum*, T6 = alginate beads of *T. harzianum* + *F. oxysporum*, T7 = only *F. oxysporum* and T8 = Carbendazim (1000 ppm) + *F. oxysporum*. The seeds were surface sterilized and grown in plant growth chamber (8 h dark/16 h light, 65–75% relative humidity, 25 °C) for 3 weeks. After 3 weeks, seedlings were uprooted cautiously and transferred to pots (1 seedling per pot). The treatments (10 g pot^−1^) were added at the time of seedling transfer. Seven days after sowing, the plants were infected with 10 ml of fungal spore suspension of pathogen (10^6^ spore ml^−1^). Each treatment comprised of 9 replicates and all pots were kept in a totally randomized block design. After 6 months, destructive sampling was performed to analyze the effect of treatments on plant growth parameters and disease control. Fresh weight, fruit yield, root and shoot lengths were measured. For determination of disease reduction and disease severity index, following scores were assigned to plants on the basis of severity level: 1 = no symptom of disease; 2 = plant showed slight infection of yellow leaves and physical wilting of 1–20%, 3 = plant showed yellowing leaves and wilting 21–40%, 4 = plant showed yellowing leaves and wilting 41–60%, 5 = severe infection with plant showed yellowing leaves and wilting 61–80%, and 6 = the whole plant leaves became yellow, 100% of wilted leaves or the plant died^15^. Further, disease reduction (%) and disease severity index (%) were calculated using the formulae mentioned by Song et al.^49^.

### Hydrolytic enzymes

Quantitative estimation of hydrolytic enzymes viz., protease, chitinase and β-1,3-glucanase was done as per the protocols mentioned by Alnahdi, Zarei, and Ting and Chai, respectively^42,50,51^. For protease, one ml culture of each microbe was inoculated in 50 ml of the minimal media, supplemented with 0.5% casein. Triplicates were prepared for each isolate. The process was repeated for chitinase and β-1, 3-glucanase with the difference that colloidal chitin and laminarin was used, respectively, in place of casein as the substrate. The cell free supernatant was taken as crude enzyme extract, and the reducing sugar was spectrophotometrically determined at 530 nm. One unit of enzyme is described as the amount of enzyme that released 1 μg of reducing sugar per ml per minute. Similarly for protease, the quantity of enzyme that releases 1 μg of tyrosine per ml per minute was stated as one unit of protease in standard conditions.

### Activity of compounds produced by biocontrol agents in culture filtrate

The bacterial and fungal strains were cultured as described in section ‘Formulation development’. The filtered broth was centrifuged at 10,000 rpm (4 °C) for 10 min. The collected supernatant was filtered through a Millipore filter (0.45 μm pore size) to obtain cell free culture filtrate. Cell free culture filtrate of biocontrol agents was incorporated in molten PDA in order to make final concentrations of filtrate as 10, 20, 30 and 40% (v/v) in the medium^48^. PDA without any culture filtrate served as control. A 5 mm fungal disc of pathogen was placed at the centre of petriplate with amended PDA. The plates were incubated for 7 days at 28 °C. After incubation, mycelial diameter was measured and percentage inhibition was calculated^52^. Further for the extraction of compounds, 5 and 7 days old bacterial and fungal cultures, respectively, were filtered through Whatman no. 1 filter paper. Organic solvent was added to culture filtrate in the ratio of 1:1 (v/v) and shaken for 5 min. Subsequently the aqueous layer and solvent were separated by solvent fractioning^39^. Solvents with different polarities were used for the extraction of compounds viz., hexane (non-polar), ethyl acetate (medium polar) and butanol (polar). The solvent fraction was collected separately and Rotary Evaporator (Buchi, Switzerland) was used for the evaporation of collected fractions. The crude extract was dissolved in DMSO (neutral solvent) for agar well diffusion assay^53^. PDA plate was inoculated with 5 mm mycelial disc of fungal pathogen at one end of the plate. On the other end, a 5 mm well was bore using a sterile cork borer under aseptic conditions. Then, 100 μl crude extract (with the same concentration of 50 mg/ml) was added in the wells. Plates with only DMSO in the wells served as control. Subsequently, the plates were incubated for 7 days at 28 °C, and observed for mycelial inhibition. The extract with maximum inhibition was further subjected to GC–MS for the identification of volatile compounds as per the conditions mentioned in previous study^54^.

### Isolation of bioactive compounds

The solvent screened above section was used in in vitro antagonism assay and for the extraction of non-volatile compounds from culture filtrate of biocontrol agents. Then, the separation of the compounds in microbial crude extract of the biocontrol agents was performed using TLC (TLC silica gel 60 F_254_, 20 × 20 cm aluminium sheets, Merck)^55^. Ten microlitre of crude extract was spotted on the TLC strip (2 cm × 10 cm) at a distance of 1.5 cm from the bottom (Fig. S10). The developing solvent consisted of an organic solvent or a combination of solvents. For the standardization of developing solvent system, six solvents of different polarities viz., ethyl acetate (EA), petroleum ether (PE), methanol (MeOH), acetone (A), hexane (H) and chloroform (CHCl_3_) and their combinations were tested for both biocontrol agents. Hundred millilitres of each solvent was taken in closed developing chamber and allowed to saturate the atmosphere of the chamber. After that, spotting was done on the marked originating point (Fig. S10), and left at room temperature to evaporate the residual solvent. After evaporating the solvent, the TLC strip was placed carefully in the chamber so that the spot remained above the solvent level. It was kept in the chamber till the solvent front moved up to the marked line. Then, the developed TLC strip was left for drying at 40 °C to evaporate the residual solvent. The developed and dried TLC strips were visualized under visible light and ultraviolet (UV) light viz., long wavelength (365 nm) and short wavelength (254 nm) using an Ultra Violet Fluorescence Analysis Cabinet (JPC Lab Solution, India). After separating the chemical constituents of crude extract on TLC plate, pathogen or test microorganism was exposed to all compounds on the same, by direct bioautography^56^. Ten days old culture of fungal pathogen was added to PDB to maintain density of 10^6^ spores ml^−1^ and sprayed on the developed TLC plates. After spraying, the TLC strip was kept in a petriplate with moistened blotting paper placed on the surface. The procedure was performed in laminar airflow under aseptic conditions. Then, the plates were placed for overnight incubation in dark at 28 °C under humid conditions. The TLC strips were then sprayed with a solution of 3-(4,5-dimethylthiazol-2-Yl)-2,5-diphenyltetrazolium bromide (MTT) dye (5 mg/ml) (SRL, India) and again kept for incubation of 2 days at 28 °C. The TLC strips were observed for the white inhibition zone and retention factor (Rf) was calculated for the desired bands using the following formula:

$$Rf = \frac{Distance \;of\; compound}{{Distance\;of\;solvent}}$$

The band of interest was scrapped along with silica after running the same sample a number of times (8 to 10) to increase the concentration of the compound. DMSO was added to the scrapped silica in the ratio of 5:1 (v/w). It was kept overnight under shaking conditions. Thereafter, it was centrifuged for 10 min at 8000 rpm and supernatant was filtered using Millipore filter of 0.45 μm pore size. The filtered extract was then subjected to UPLC-MS for identification. The UPLC-MS analysis was carried out using Waters Acquity UPLC system (Waters Corp., Milford, USA) as per the conditions mentioned in previous study^54^.

### Statistical analysis

The data was obtained in triplicates and statistical analysis was carried out by one-way analysis of variance (ANOVA) using SPSS (version 17.0). Tukey’s HSD test was performed to test the significance of difference. *P* values < 0.05 were considered as statistically significant. For RSM, analysis of variance (ANOVA) was used for the statistical analysis of the model. Fisher’s test value was used for the determination of statistical significance of the model equation, and the proportion of variance described by the model was given by the multiple coefficient of determination, R squared (R2) value. For each response, the quadratic models were signified in the form of contour plots (3D) and response surface curves were also made. The exact mass of different ions obtained from MS fragmentation was identified using metabolite search tool of METLIN (https://metlin.scripps.edu). The tolerance on the mass of precursor ion was taken as 10 ppm.

### Ethical approval

This article does not contain any study with human participants or animals performed by any of the authors.

## Supplementary Information

Supplementary Information.
