# Supplementary material for: Development of next-generation formulation against Fusarium oxysporum and unraveling bioactive antifungal metabolites of biocontrol agents
Source: Sci Rep. 2021 Nov 24;11:22895. doi: 10.1038/s41598-021-02284-1 (PMC8613265; doi:10.1038/s41598-021-02284-1)
Supplement: Supplementary file 1 — Supplementary Information. [file 41598_2021_2284_MOESM1_ESM.docx]

**Table S1.** Response for water dispersible granules developed with combination of different substrates, bulking agent, dispersing agent and wetting agent.

| **Combination** | **Substrate** | **Bulking agent** | **Dispersing agent** | **Wetting agent** | **Response** |
| --- | --- | --- | --- | --- | --- |
| I | Silica | China clay | Napthalene sulphonate | Sodium lauryl sulphate | Very low dispersion rate |
| II | Maize flour | China clay | Napthalene sulphonate | Sodium lauryl sulphate | No dispersion |
| III | Wheat flour | China clay | Napthalene sulphonate | Sodium lauryl sulphate | No dispersion |
| IV | Wheat bran | China clay | Napthalene sulphonate | Sodium lauryl sulphate | No dispersion |
| V | Silica | Vermiculite | Napthalene sulphonate | Sodium lauryl sulphate | No dispersion |
| VI | Talcum powder | Talcum powder | Alginic acid | Acacia gum | Dispersion |
| VII | Talcum powder | Talcum powder | Micro-crystalline cellulose | Acacia gum | Very low dispersion rate |

**Table S2.** Inhibition of biocontrol agents with chemicals used in preliminary development of water dispersible granules (± standard deviation, *n*= 3)

| **Reagent** | **Percentage inhibition on bacterial colonies (in %)** | **Percentage inhibition on radial mycelial growth of *Trichoderma harzianum* (in %)** |
| --- | --- | --- |
| Napthalene sulphonate (1%) | No inhibition | No inhibition |
| Napthalene sulphonate (2%) | 25.7 ± 2 | 29.4 ± 1.9 |
| Sodium lauryl sulphate (1%) | No inhibition | No inhibition |
| Sodium lauryl sulphate (2%) | No inhibition | No inhibition |
| Sodium lauryl sulphate (3%) | No inhibition | No inhibition |
| Sodium lauryl sulphate (4%) | No inhibition | No inhibition |
| Sodium lauryl sulphate (5%) | No inhibition | No inhibition |
| Sodium lauryl sulphate (6%) | 38.4 ± 2.6 | 34.2 ± 2.3 |
| Sodium lauryl sulphate (7%) | 42.3 ± 3.7 | 39.1 ± 5.4 |

**Table S3.** ANOVA analysis for (a) response 1 [wetting time (s)], (b) [dispersing time (s)] and (c) response 3 [Suspensibility (%)]

**(a)**

| **Source** | **Sum of squares** | **df** | **Mean square** | **F-value** | **P-value** | **Remark** |
| --- | --- | --- | --- | --- | --- | --- |
| **Model** | 22.54 | 5 | 4.51 | 40.50 | < 0.0001 | Significant |
| A-Acacia gum | 0.0011 | 1 | 0.0011 | 0.0100 | 0.9231 |  |
| B-Alginic acid | 9.28 | 1 | 9.28 | 83.39 | < 0.0001 |  |
| AB | 0.9801 | 1 | 0.9801 | 8.81 | 0.0209 |  |
| A² | 11.95 | 1 | 11.95 | 107.38 | < 0.0001 |  |
| B² | 1.04 | 1 | 1.04 | 9.30 | 0.0186 |  |
| Residual | 0.7791 | 7 | 0.1113 |  |  |  |
| **Lack of Fit** | 0.6416 | 3 | 0.2139 | 6.22 | 0.0549 | Not significant |
| Pure error | 0.1375 | 4 | 0.0344 |  |  |  |
| Cor total | 23.32 | 12 |  |  |  |  |

**(b)**

| **Source** | **Sum of squares** | **df** | **Mean square** | **F-value** | **P-value** | **Remark** |
| --- | --- | --- | --- | --- | --- | --- |
| **Model** | 112.54 | 5 | 22.51 | 20.95 | 0.0004 | Significant |
| A-Acacia gum | 47.28 | 1 | 47.28 | 44.00 | 0.0003 |  |
| B-Alginic acid | 12.39 | 1 | 12.39 | 11.53 | 0.0115 |  |
| AB | 0.7482 | 1 | 0.7482 | 0.6963 | 0.4316 |  |
| A² | 46.48 | 1 | 46.48 | 43.26 | 0.0003 |  |
| B² | 10.52 | 1 | 10.52 | 9.79 | 0.0166 |  |
| Residual | 7.52 | 7 | 1.07 |  |  |  |
| **Lack of Fit** | 5.95 | 3 | 1.98 | 5.04 | 0.0761 | Not significant |
| Pure error | 1.57 | 4 | 0.3935 |  |  |  |
| Cor total | 120.06 | 12 |  |  |  |  |

**(c)**

| **Source** | **Sum of squares** | **df** | **Mean square** | **F-value** | **P-value** | **Remark** |
| --- | --- | --- | --- | --- | --- | --- |
| **Model** | 116.67 | 5 | 23.33 | 14.62 | 0.0014 | Significant |
| A-Acacia gum | 0.9013 | 1 | 0.9013 | 0.5646 | 0.4769 |  |
| B-Alginic acid | 2.31 | 1 | 2.31 | 1.45 | 0.2679 |  |
| AB | 40.90 | 1 | 40.90 | 25.62 | 0.0015 |  |
| A² | 66.73 | 1 | 66.73 | 41.80 | 0.0003 |  |
| B² | 11.98 | 1 | 11.98 | 7.50 | 0.0290 |  |
| Residual | 11.17 | 7 | 1.60 |  |  |  |
| **Lack of Fit** | 4.72 | 3 | 1.57 | 0.9746 | 0.4878 | Not significant |
| Pure error | 6.46 | 4 | 1.61 |  |  |  |
| Cor total | 127.85 | 12 |  |  |  |  |

**
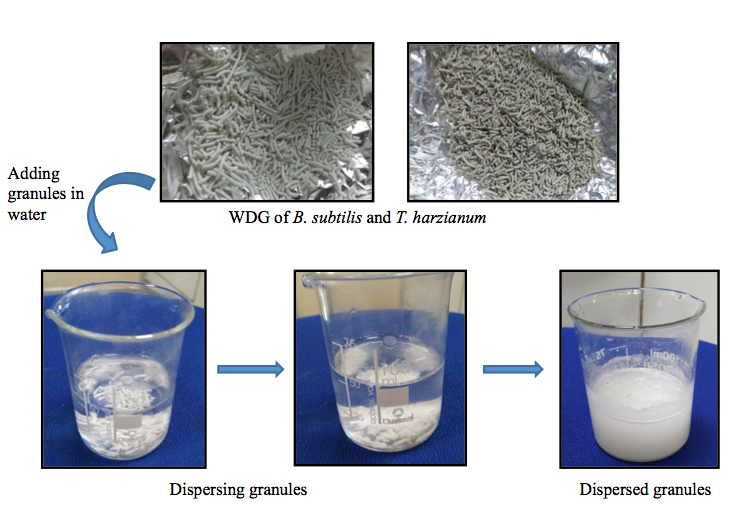
**

**Fig. S1** Dispersion of water dispersible granules (WDG) developed with optimized concentration of wetting agent and dispersing agent


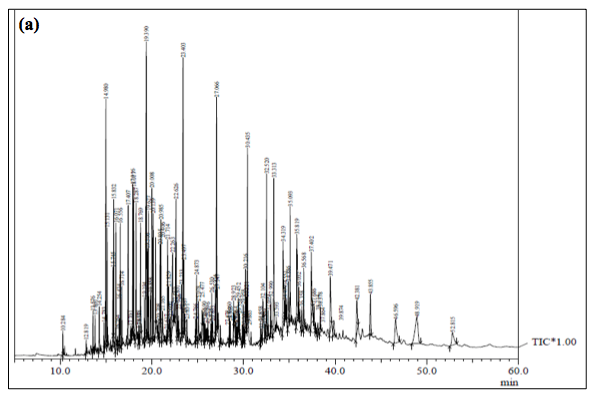


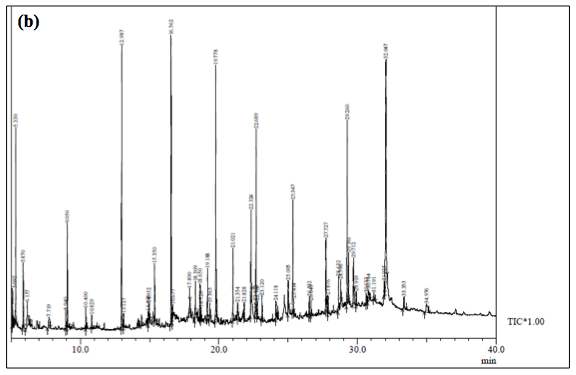


**Fig. S2** Chromatogram of volatile organic compounds produced by (a) *Bacillus subtilis* and (b) *Trichoderma harzianum* extracted with ethyl acetate and identified by GC-MS with their retention time.

**
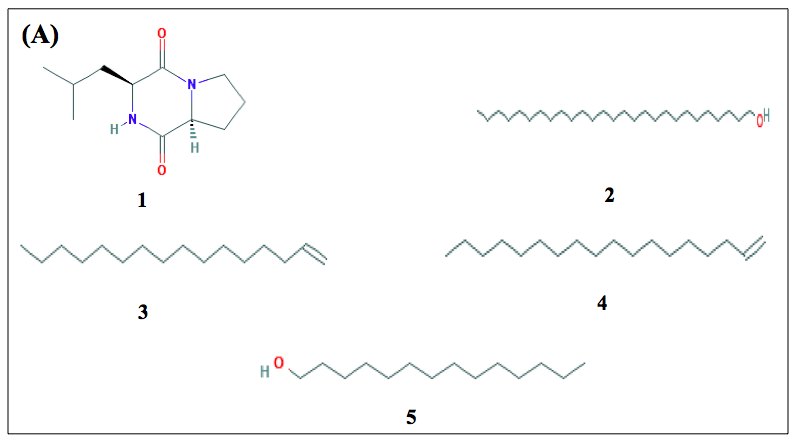
**

**
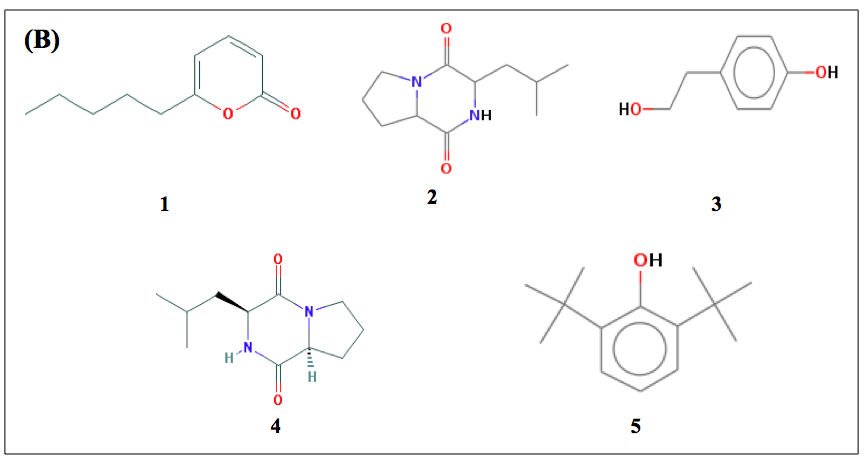
**

**Fig. S3** Structures of major compounds in culture filtrate of (A) *Bacillus subtilis* and (B) *Trichoderma harzianum* identified by GC-MS in ethyl acetate extract. For name of the compound refer to **Table 4**.

**
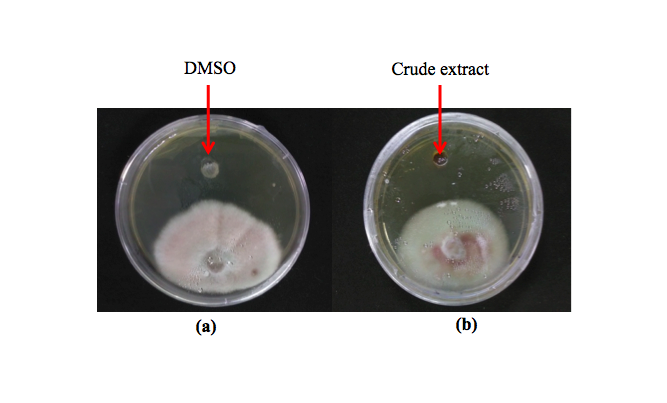
**

**Fig. S4** Antifungal activity of ethyl acetate extract (dissolved in DMSO) of *Bacillus subtilis* against *Fusarium oxysporum* in agar well diffusion assay with (a) control (blank DMSO), and (b) crude extract of ethyl acetate.

***
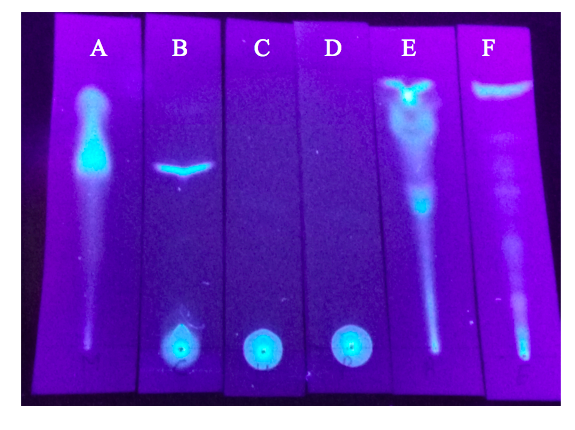
***

**Fig. S5** Thin layer chromatography for standardization of solvent system for ethyl acetate crude extract of *Bacillus subtilis* where A = methanol, B = chloroform, C = hexane, D = petroleum ether, E = acetone, and F = ethyl acetate.

***
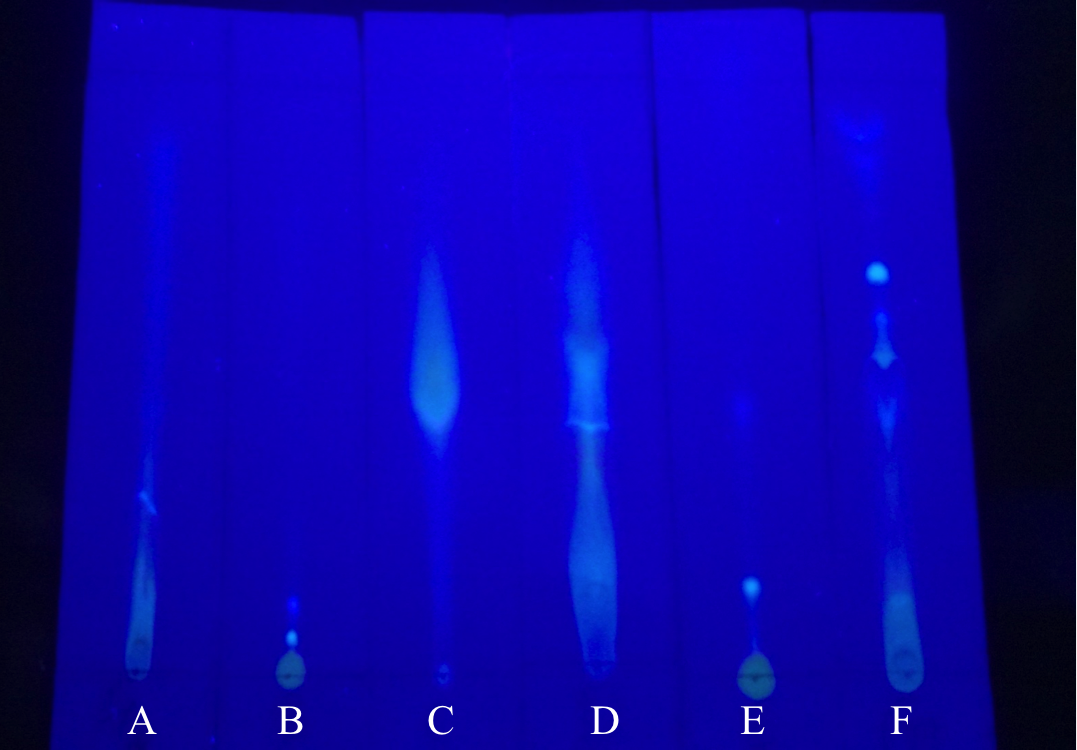
***

**Fig. S6** Thin layer chromatography for standardization of solvent system for ethyl acetate crude extract of *Trichoderma harzianum* where A = ethyl acetate, B = petroleum ether, C = methanol, D = acetone, E= hexane, and F = chloroform.


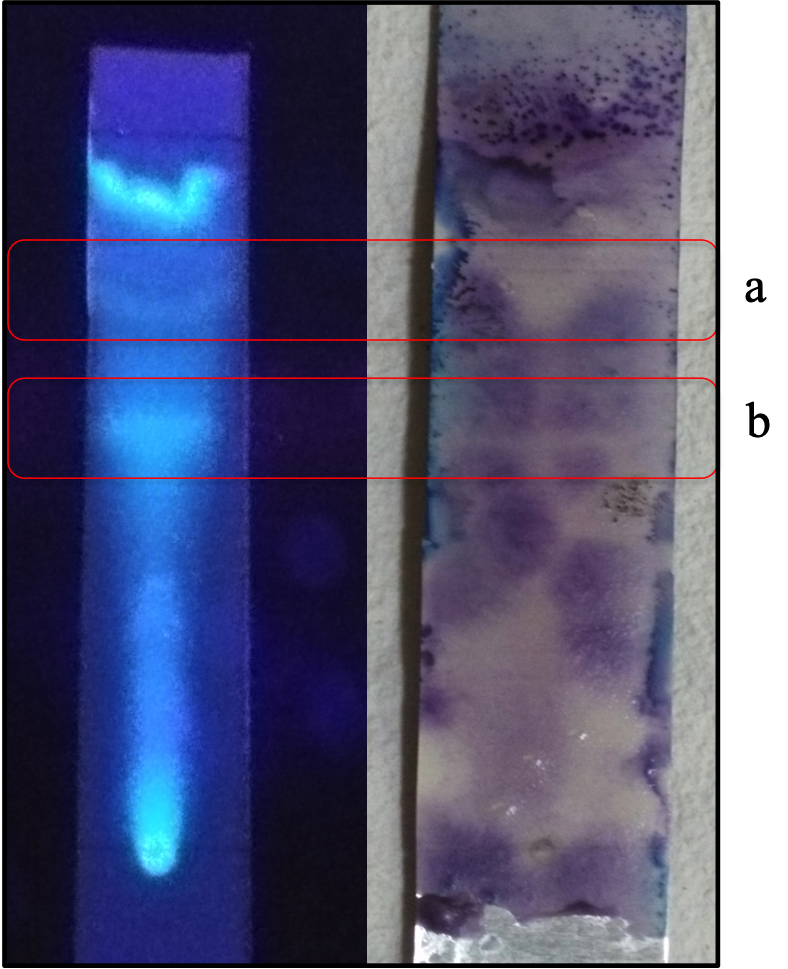


**Fig. S7** Direct bioautography for *Bacillus subtilis* with ethyl acetate as the mobile phase for developing TLC. ‘a’ and ‘b’ depict white inhibition zone with retention factor of 0.475 and 0.625, respectively.

***
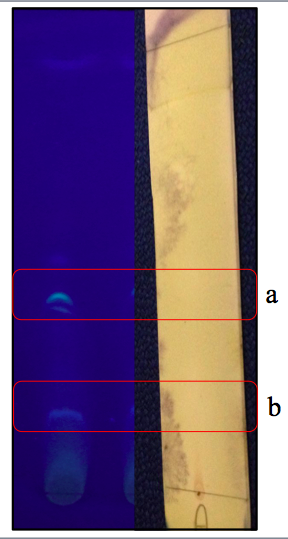
***

**Fig. S8** Direct bioautography for *Trichoderma harzianum* with mixture of chloroform and petroleum ether (8:2) as the mobile phase for developing TLC. ‘a’ and ‘b’ denote white inhibition zone with retention factor of 0.175 and is 0.4125, respectively.


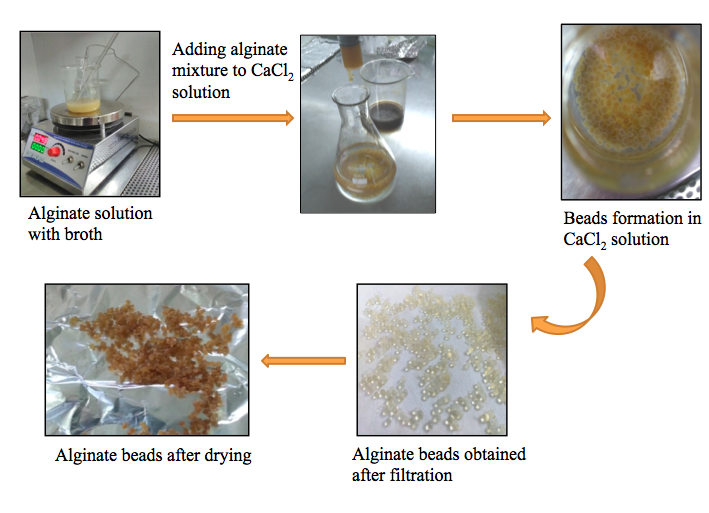


**Fig. S9** Schematic diagram showing the steps for development of alginate beads

**Table S4.** Experimental design employed in the response surface methodology (RSM) using a central composite design matrix for increasing efficiency of water dispersible granules

| **Run** | **Variable A**  **Wetting agent (g)** | **Variable B**  **Dispersing agent (g)** |
| --- | --- | --- |
| 1 | 0.76 | 10 |
| 2 | 5 | 10 |
| 3 | 2 | 16 |
| 4 | 8 | 16 |
| 5 | 5 | 10 |
| 6 | 2 | 4 |
| 7 | 9.24 | 10 |
| 8 | 5 | 18.49 |
| 9 | 5 | 10 |
| 10 | 8 | 4 |
| 11 | 5 | 10 |
| 12 | 5 | 1.51 |
| 13 | 5 | 10 |


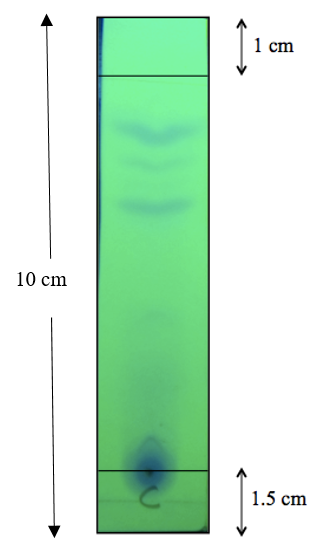


**Fig. S10** Illustration of a developed thin layer chromatography
